# Compaction Behavior and Damage Constitutive Model for Porous Cement Mortar under Uniaxial Cyclic Loads

**DOI:** 10.3390/ma15238302

**Published:** 2022-11-22

**Authors:** De-Hang Liu, Yue Qin, Li Zhuo, Jian-Feng Liu, Zhao-Qiang Zheng, Jian-Liang Pei, Huai-Zhong Liu

**Affiliations:** State Key Laboratory of Hydraulics and Mountain River Engineering, College of Water Resources and Hydropower, Sichuan University, Chengdu 610065, China

**Keywords:** porous cement mortar, constitutive model, void compression stage, irreversible deformation, damage variable

## Abstract

The void compression stage causes porous cement mortar to present special mechanical properties. In order to study the compaction behavior and the damage evolution of the porous material, cement mortar specimens with an average porosity of 26.8% were created and cyclic uniaxial compression tests were carried out. The irreversible strain accumulated in the tests was obtained by cyclic loading and unloading. As the secant modulus of the porous cement mortar increases with stress in the pre-peak deformation stage, its damage variable is defined according to the accumulated irreversible strain instead of modulus degradation. The strain-based damage indicator fitted with the damage evolution law is characterized by linear accumulation at the beginning and has an acceleration rate of about 0.3 in the pre-peak deformation stage, and the damage value converges to 1 at failure. Based on the Weibull distribution, a constitutive damage model of porous cement mortar is improved by considering both the damage evolution during the plastic deformation stage and the mechanical behavior in the compaction stage. The theoretical envelope curves obtained by the constitutive model are in good agreement with the experimental envelope curves of cyclic uniaxial compression in the compaction and pre-peak stages, and the average absolute error is about 0.54 MPa in the entire pre-peak stage, so the proposed damage constitutive model can characterize the damage-induced mechanical properties of porous cement mortar in the compaction and pre-peak stages.

## 1. Introduction

As a special building material, porous cement mortar has a higher porosity than other materials, making it applicable in specific situations, such as heat preservation, masonry structure, damping measures, and isolation [1,2,3]. Therefore, it is of great significance to comprehend the mechanical properties of porous cement mortar and to characterize the damage-induced mechanical behavior of this material under cyclic loads using a proper constitutive model.

In the past 20 years, studies have been carried out to elucidate the mechanical properties of cement mortar, and several constitutive models of cement mortar have been proposed. Yang et al. [4] and Chen et al. [5] proposed a constitutive damage model of cement as well as cement-based materials through compressive experiments, including under wet-dry cyclic conditions. Kakavas et al. [6] and Chidiac and Mahmoodzadeh [7] established constitutive flow models of fresh mortar based on the same assumption that the flow can be represented by three interactions: static interaction, dynamic interaction, and collision. Tan et al. [8] researched initial damage and proposed a constitutive model based on the energy theory. Li and Ou [9] proposed a constitutive damage model of cement mortar based on three-point bending test results. Zhou and Chen [10] conducted Split Hopkinson Pressure Bar (SHPB) tests and proposed a dynamic damage constitutive model of cement mortar. Xie et al. [11] concluded that the peak strength increases with the increase in the strain rate in a compressive experiment on cement asphalt mortar. Andronikos et al. [12] proposed an electrical method to sense damage to the cement mortar. Zhu et al. [13] elucidated the damage evolution and dynamic performance of cement asphalt mortar under dynamic vehicle load and developed a statistical damage constitutive model for numerical simulation. Moreover, Chen et al. [14] took a thermodynamic approach to comprehend the elastoplastic behaviors of heat-treated mortar and proposed a constitutive damage model.

Moreover, porous materials behave in a way that is rather different from dense materials. In this field, remarkable progress has been achieved in the study of other porous materials, such as porous rocks [15], porous metal [16], and other porous materials [17,18,19,20]. Nunziato and Cowin [21] and Green [22] proposed theoretical models of porous materials from the perspective of the elasticity–plasticity theory. Hom and McMeeking [23] elucidated the process of void growth in porous elastoplastic materials using finite element simulations. Moreover, many scholars have found that the mixture ratios of different substances can significantly affect the porosity of cement materials. Joaquín Abellan-García [24] studied the relationships between the ultra-high-performance concrete (UHPC) dosage and its resulting properties and found that an excess of HRWR may result in a high internal porosity of UHPC. Mohamed et al. [25] conducted tests on three asphalt cements with different percentages of carbon nanotubes (CNTs), and the results determined that modifying asphalt cement with CNTs decreased its penetration. Afonso et al. [26] added natural fiber extracted from the crown of pineapple to mortar and discovered that mortars containing treated fibers exhibit a gradual decrease in the air-incorporated concentration as fiber incorporation increases. However, the progressive compaction behavior at the beginning of loading, which distinguishes porous materials from dense materials, has rarely been considered in modeling the mechanical behaviors of porous cement mortar under compression loads.

The stress–strain relationship in the compaction stage plays a very important role in the irreversible compaction mechanism, which almost occupies the entire pre-peak deformation behavior of porous cement mortar. However, it was usually neglected in the constitutive modeling of porous cement mortar. Thus, its stress–strain relationship induced by the irreversible compaction mechanism is modeled by introducing damage mechanics in this study. Although the damage evolution of porous cement mortar during the loading process was widely studied, the damage variable was inversely determined after fitting the stress–strain curve by a constitutive model that involves the damage variable. In this study, the damage variable is defined by the irreversible strain directly measured by the cyclic compression test. Thus, uniaxial cyclic loading–unloading compression tests were conducted to measure the irreversible evolutionary deformation, and the classic Weibull distribution function was employed to characterize the evolution of damage. A constitutive damage model was finally established to characterize the irreversible compaction behavior of the porous cement mortar. The investigation and modeling of cement mortar’s deformation behavior are of great significance in evaluating the safety of masonry structures and enriching the works in the research community of cement mortar.

## 2. Materials and Methods

### 2.1. Specimen Preparation and Experimental Devices

**Figure 1:**
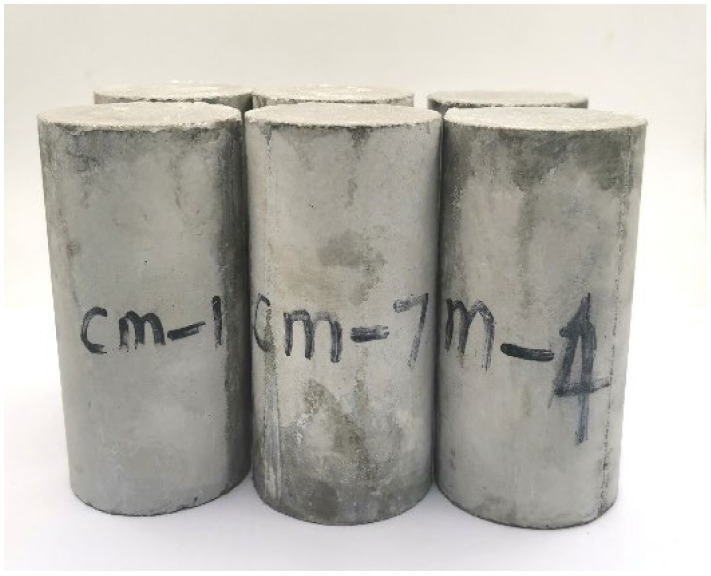
Cement mortar specimens for mechanical testing.

The porous cement mortar was made in a ratio of water: cement: standard sand: kaolin = 2:3:4:1. To increase the porosity of the cement mortar, an air-entraining agent with a mass ratio of 0.78 g per kilogram was added to the mortar slurry. The mixed proportion and the addition of the air-entraining agents had an important influence on the porosity, and the standard sand contributed to the strength of the specimens. After being conserved in a standard curing room at a temperature of 20 ± 5 °C and relative humidity of 95% for 28 days, the specimens were processed into cylinders with a diameter of 50 mm and a height of 100 mm according to the method recommended by the International Society for Rock Mechanics (ISRM), as shown in Figure 1. The specimens were divided into two groups. One group of specimens were used for compression tests, and the other specimens were used for porosity measurement. The basic information of the specimens prepared for compression tests is listed in Table 1. The detailed process of porosity measurement is described as follows. First, the specimens were dried in an oven at a temperature of 105 °C for 48 h and then weighed immediately. Second, the specimens were weighed again immediately after immersed in distilled water under vacuum conditions for another 48 h. Finally, the mass difference of the specimen before and after saturation and the volume of the specimen were used to calculate the porosity. The porosity was determined as:

(1) $$\rho =\frac{m_{s}-m_{d}}{V}$$

where *ρ* is the porosity, *m*_s_ and *m*_d_ are the masses of the oven-dried and water-saturated specimen, respectively, and *V* is the volume of the specimen. The average porosity of the studied cement mortar is 26.8%.

**Figure 2:**
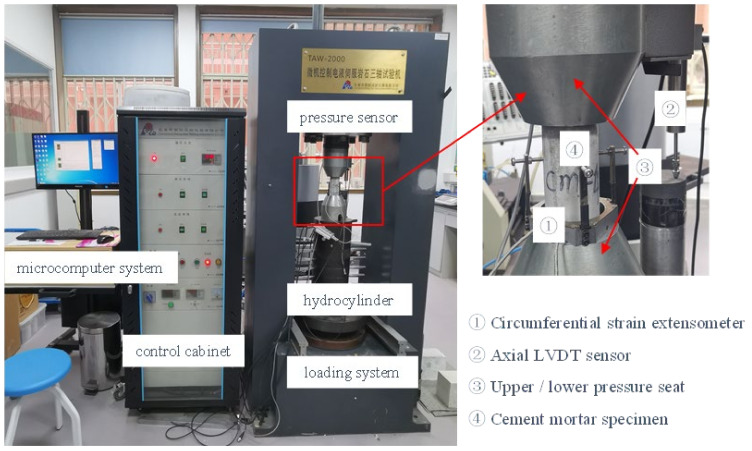
TAW-2000 microcomputer-controlled electro-hydraulic servo rock triaxial testing system.

The experiments were conducted using the TAW-2000 microcomputer-controlled electro-hydraulic servo rock triaxial testing system (Chaoyang Testing Instruments, Changchun, China) at Sichuan University, as shown in Figure 2. The maximum axial pressure was 2000 kN, and the maximum confining pressure was 100 MPa. The axial and circumferential strains were measured by a linear variable displacement transducer (LVDT) (Chaoyang Testing Instruments, Changchun, China) and a circumferential strain extensor, with measurement ranges of 0~5 mm and 0~3 mm, respectively.

### 2.2. Test Process

**Figure 3:**
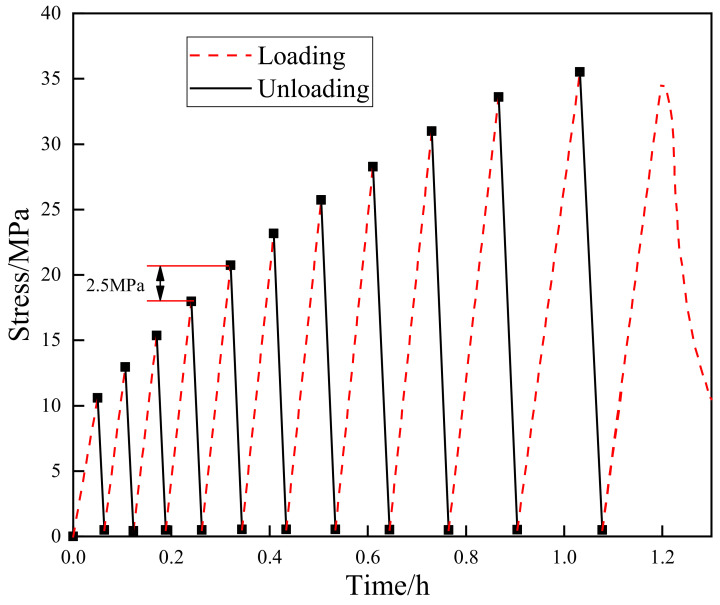
Tests stress path diagram.

In the present study, two kinds of experiments were conducted: (1) uniaxial monotonic compression tests to determine the basic mechanical properties of the material, and (2) uniaxial cyclic loading-unloading tests to obtain the evolution of irreversible strain. The cyclic loading path was determined based on the following principles: the axial stress should be unloaded to almost zero to obtain irreversible strain, and the loading rate should be small enough to avoid the dynamic effect. As shown in Figure 3, the uniaxial compression tests were conducted in axial deformation control mode at an axial deformation rate of 0.06 mm/min, and the cyclic uniaxial compression tests were conducted at an axial deformation rate of 0.06 mm/min during loading and at an axial stress rate of 0.25 MPa/s during unloading. The detailed loading and unloading process implemented during the cyclic compression tests was as follows: In the first stage, the specimen was loaded to 10.00 MPa at an axial deformation rate of 0.06 mm/min in order to avoid sudden destruction. Then, it was unloaded to 0.50 MPa at an axial stress rate of 0.25 MPa/s in order to obtain accurate unloading stress. In the subsequent loading–unloading cycles, the upper limit of the axial stress was increased by 2.50 MPa each time, whereas the lower limit of unloading was kept invariable. When the specimen was loaded to the peak stress, the axial stress was immediately unloaded to 0.50 MPa, and the specimen was then loaded to failure at an axial deformation rate of 0.06 mm/min. All of the test processes were conducted according to the method recommended by the International Society for Rock Mechanics (ISRM).

## 3. Analysis of Stress–Strain Curves

### 3.1. Stress–Strain Curves

**Figure 4:**
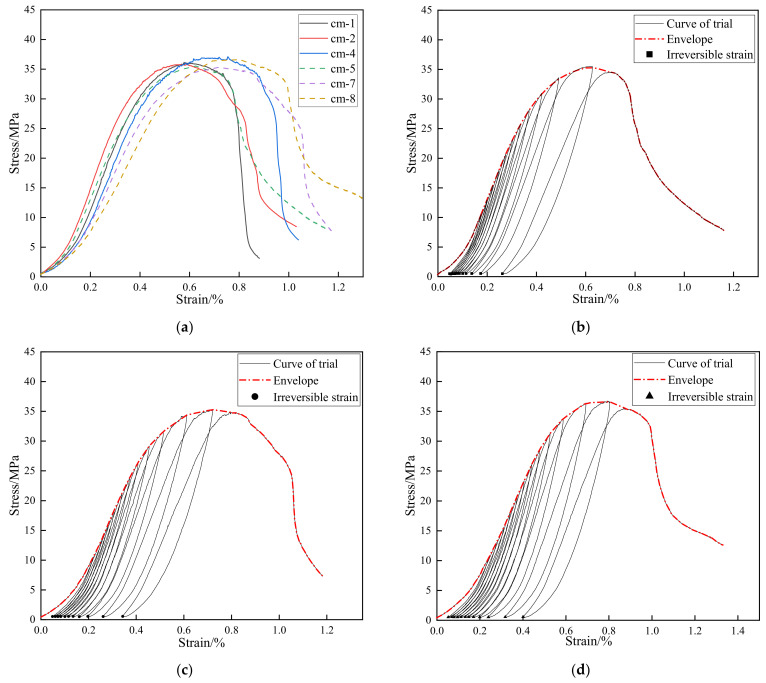
Stress–strain curves of conventional uniaxial compression tests and cyclic loading and unloading tests: (**a**) all specimens; (**b**) cm-5; (**c**) cm-7; and (**d**) cm-8.

In Figure 4a, the dashed curves are the envelope lines of the cyclic loading stress–strain curves. In the pre-peak stage, the axial stress–strain curves go through a compaction stage and then increase linearly for a period of time, followed by a distinct yielding stage towards the peak point. In the post-peak stage, the envelope curves present an evolutionary characteristic of “gentle–steep–gentle”. More specifically, the curves drop rapidly after experiencing a slow strain-softening stage and then enter the residual stage until the specimen collapses. The solid lines are the stress–strain curves of the cement mortar under uniaxial monotonic compression loading. In the pre-peak stage, the curves are similar to the stress–strain curves of the cyclic loading ones, but Young’s modulus increases slightly. In the post-peak stage, the stress–strain curves drop rapidly after a slight stress reduction and reach the residual state at failure.

As can be seen from Table 2, the average values of the peak strength and Young’s modulus in the monotonic compression tests are 36.35 MPa and 10.21 GPa, respectively, whereas the average peak stress of the cyclic loading and unloading envelope is 35.81 MPa, and the average Young’s modulus is 8.78 GPa. Despite slight decreases in the peak strength and Young’s modulus, the envelope of the cyclic loading–unloading stress–strain curves are close to the stress–strain curves under monotonic compression loads in shape, as shown in Figure 4. Therefore, we considered the influence of cyclic loading–unloading history to be limited to the strength properties, and the irreversible strains measured in cyclic loading-unloading tests could approximately represent the irreversible deformation in monotonic compression tests.

According to Figure 4, the envelopes of the cyclic compression curves and the stress–strain curves of the monotonic compression tests have extremely strong similarities in movement. In the pre-peak stage, both the cyclic compression and monotonic compression curves show a similar trend of “compaction–linearity–yielding”. In the post-peak stage, both of them show a similar softening rate.

**Figure 5:**
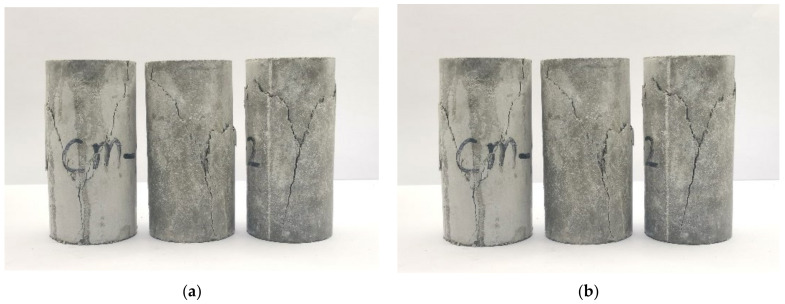
Failure patterns of specimens: (**a**) uniaxial monotonic compression tests; (**b**) uniaxial cyclic loading and unloading tests.

As shown in Figure 5, the failure pattern of cement mortar specimens under uniaxial cyclic loading–unloading is characterized by two intersected shear fracture surfaces, which is similar to that in monotonic compression tests. Different from materials with compact structures, the porous internal structure of the cement mortar allows more amount of shearing dislocation between particles, and the microcracks are prone to expand along the boundary of the aggregates due to the weak bonds between particles, which is quite similar to the failure modes of some porous materials [27,28].

The difference in porosity causes the change in the strength of cement mortar. The Balshin model describes a relationship between porosity and compressive strength of materials [29,30]:

(2) $$\sigma_{m}=\sigma_{0}{\left(1-\rho \right)}^{b}$$

where *σ*_0_ is the strength at zero porosity, and *b* is the empirical constant.

**Figure 6:**
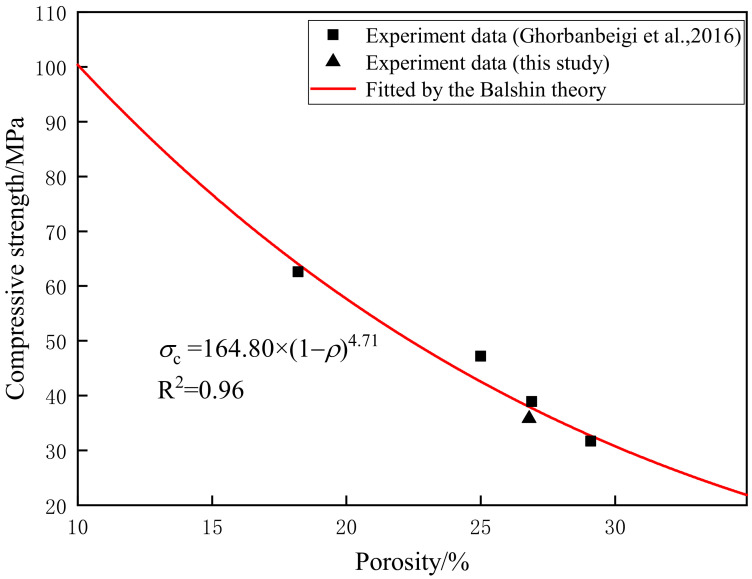
Relationship between compressive strength and porosity. Experiment data Ghorbanbeigi et al. [31].

As shown in Figure 6, the equation is in good agreement with the result of this study and other data [31], and the compressive strength of cement mortar decreases with the increase in porosity.

### 3.2. The Relationship between Irreversible Strain and Unloading Stress

**Figure 7:**
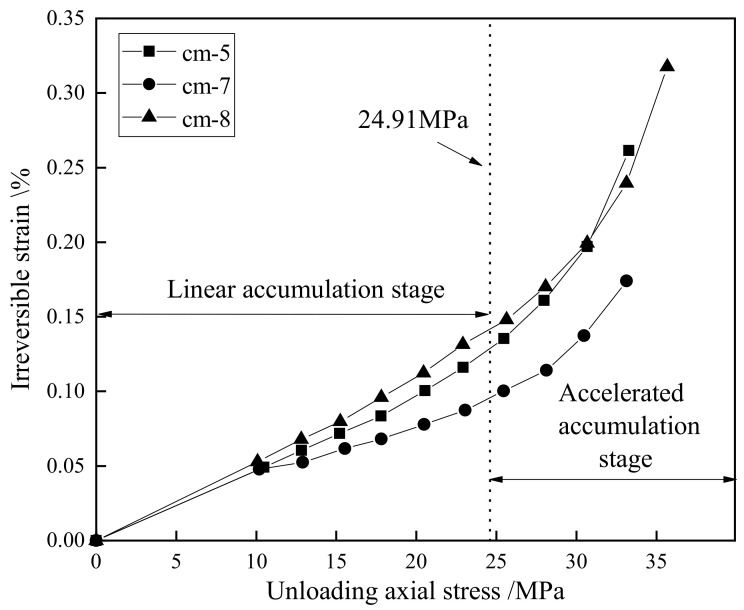
Relationship between irreversible strain and axial unloading stress.

Figure 7 shows the relationship between the irreversible strain at the terminal of unloading and the axial stress from which unloading starts, namely the unloading stress. In the study of porous materials, the irreversible strain increases during the process of cyclic loading and unloading [32,33]. The accumulated irreversible strain increases as the number of cycles increases, as seen in Table 3. It can also be seen from Figure 7 that there is a positive correlation between the irreversible deformation and the unloading stress. The curves of the accumulated irreversible strain can be divided into two stages, namely, the linear growth stage and the accelerated accumulation stage, respectively.

In the initial loading phase, the original micro-cracks were compacted, and the irreversible deformation accumulated slowly. In this study, the irreversible strain was initially recorded when the axial stress was 10 MPa, so the curves did not reflect the compaction phase. However, other studies conducting similar tests on sandstone proved the linear accumulation of the irreversible strain during the beginning of the cycle [34]. With the continuous increase in the axial stress, some closed cracks expanded again, and some new cracks initiated and developed. Additionally, the plastic deformation of the specimens accumulated continuously. When the axial unloading stress increased to approximately 25 MPa, the variation rate of the irreversible deformation increased significantly, and the specimens entered the accelerated accumulation stage, indicating that the microcracks developed unstably, and the macroscopic crack system started to form [35].

## 4. Analysis of Damage Evolution

The cyclic deformation behavior of the porous cement mortar presents two distinct inelastic mechanisms: compaction and microcracking. To better describe the impact of these mechanisms, a secant modulus *E*_u_ has been defined as

(3) $$E_{u}=\frac{\sigma_{u}-\sigma_{r}}{\varepsilon_{u}-\varepsilon_{r}}$$

where *σ_u_* and *ε_u_* are the stress and strain corresponding to the unloading point, respectively. *σ_r_* and *ε_r_* are the stress and strain corresponding to the subsequent reloading point, respectively.

**Figure 8:**
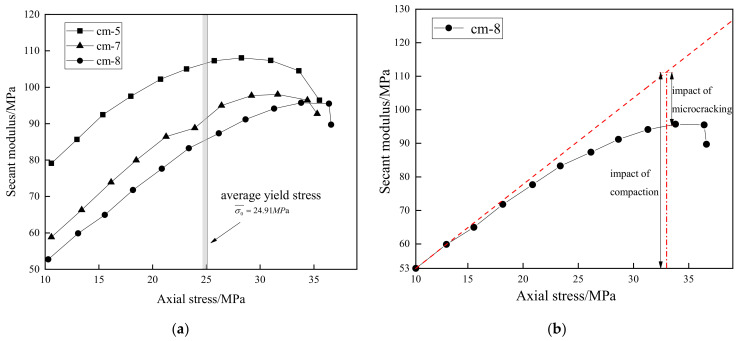
Relationship between secant modulus and axial stress: (**a**) all specimens of cyclic loading tests; (**b**) schematic analysis method for compaction and microcracking.

During the test process, compaction and microcracking caused the secant modulus to increase and decrease, respectively. The stress corresponding to the end of the linear stress–strain stage, also called yield stress, indicates that the specimen steps into the yield stage. According to the Axial stress difference (ASD) method proposed by Xie et al. [36], the average yield stress of porous cement mortar in the cyclic uniaxial compression tests was determined as 24.91 MPa. Before the yield stress is reached, the secant unloading modulus of porous cement mortar increases as the axial stress increases due to the influence of pore compaction, as shown in Figure 8a. When the axial stress surpasses the yield stress, it continues to increase for a period of time. In order to illustrate this phenomenon, a schematic analysis method is presented in Figure 8b. In the first few cycles, compaction exhibits a dominant impact on the secant modulus. The tilted dashed line that draws from the first few points of specimen 8 roughly indicates the value of the secant modulus without the impact of microcracking. The value from the intersection point of the tilted dashed line and the perpendicular dashed line to the coordinate *X*-axis can be identified as the increased modulus caused by pore compaction. The impact of microcracking that makes the secant modulus decrease can be distinguished as the difference value between the tilted dashed line and the solid line. Thus, both the compaction and microcracking mechanism affect the deformation of the yielded porous cement mortar, and the effect of compaction is stronger than that of microcracking before the peak load is reached. When the microcracking mechanism is dominant, the unloading modulus decreases.

In order to analyze the damage evolution of porous cement mortar specimens, a damage variable *D* is defined to characterize the degree of material damage. Based on different assumptions and theories, the damage variable has been defined by researchers from all over the world [37,38,39]. The secant unloading modulus has been widely used to represent the elastic deformation properties of damaged material. Based on the equivalent strain principle, the damage variable *D* can also be defined as

(4) $$D=1-E/E_{0}$$

where *E* is Young’s modulus of the damaged materials, and *E*_0_ is Young’s modulus of the undamaged materials.

Since the secant unloading modulus continues to increase after the material yields, the damage variable *D* in Equation (4) continues to drop. Thus, this definition of the damage variable is not suitable for porous materials. As the increasing irreversible strain conforms to the damage evolution of porous cement mortar specimens, it has been used to characterize the damage variable as an indicator by some researchers [40,41]. Based on the nonlinear accumulated plastic strain, the damage variable can better elucidate the damage evolution law of porous cement mortar. The damage variable *D* is defined by Xiao et al. [42] as

(5) $$D=\frac{\varepsilon_{t}}{\varepsilon_{p}}$$

where *ε*_p_ is the irreversible strain corresponding to the residual strength, and *ε*_t_ is the irreversible strain corresponding to the subsequent unloading point.

Assuming that the strengths of the porous cement mortar specimens conform to the Weibull distribution [43], the damage evolution law could be expressed as

(6) $$D=1-\exp\left[-{\left(\frac{\varepsilon }{a}\right)}^{m}\right]$$

where *a* and *m* are the parameters of the Weibull distribution, and *ε* is the strain of the specimen.

**Figure 9:**
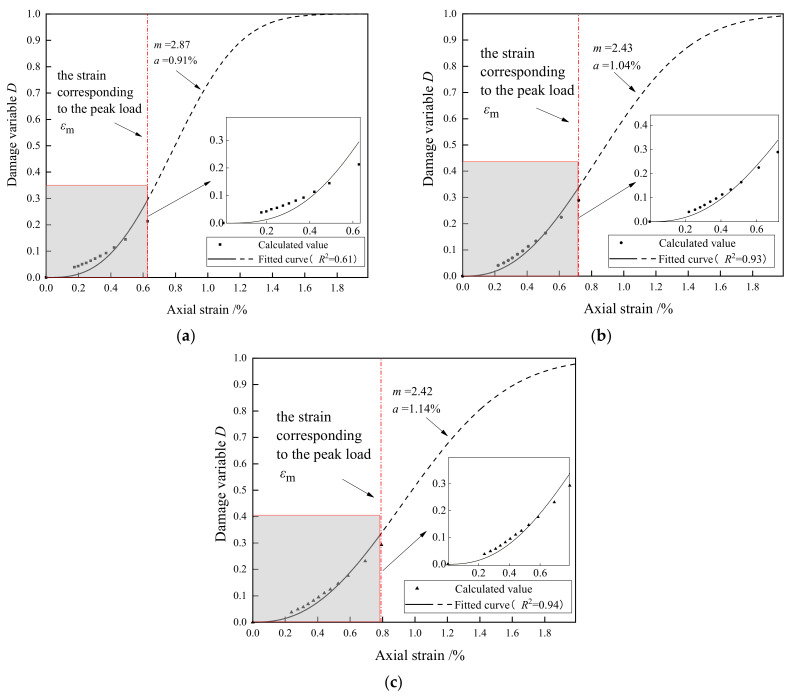
Evolution of damage variable D during the uniaxial cyclic compression process: (**a**) cm-5; (**b**) cm-7; and (**c**) cm-8.

As shown in Figure 9, the mark points are calculated according to Equation (5) and the test results of cyclic compression tests. The curves are fitted according to Equation (6) and the mark points. In the compaction stage, as the axial strain increases, the damage variable increases gradually from 0, and the growth rate is relatively slow. After the compaction stage, the damage variable increases exponentially, and the growth rate continues to increase. When the peak stress is reached, the damage growth rate reaches its maximum value. In the post-peak stage, the curves in Figure 9 show that the damage continues to accumulate, but its growth rate continues to decrease. The final value of the damage variable value converges to 1, indicating that the specimens are completely damaged. The main objective of this study is to reveal the pre-peak damage behavior of porous cement mortar for engineering purposes, something that deserves more attention from engineers than the post-peak damage behavior, so the post-peak damage behavior was not measured in the cyclic tests.

## 5. Damage Constitutive Model for Porous Cement Mortar

Lemaitre [44] proposed the equivalent strain principle, which considers the strain caused by the true stress of the damaged materials is equal to the strain caused by the equivalent stress of undamaged materials. Based on the equivalent strain principle, the constitutive relation of the damaged materials can be defined as

(7) $$\sigma =E_{0}\left(1-D\right)\varepsilon$$

**Figure 10:**
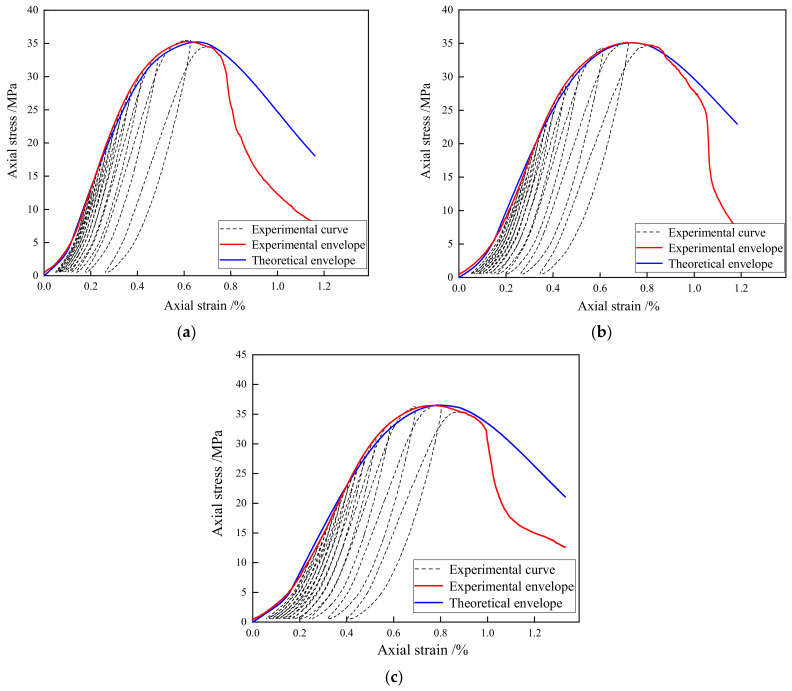
Comparison of theoretical and experimental envelope curves: (**a**) cm-5; (**b**) cm-7; and (**c**) cm-8.

As a porous material, cement mortar presents a strong void compression effect under cyclic uniaxial compression conditions, as observed in Figure 10. Thus, it is necessary to take the void compression stage into consideration when proposing a constitutive model of porous cement mortar. Based on the Weibull distribution model, Bian et al. [45] proposed a constitutive relationship of rock under the influence of void compression, which can be expressed as

(8) $$\sigma =\left\{\begin{array}{ll} E\varepsilon \left\{1-\exp\left[-{\left(\frac{\varepsilon }{b}\right)}^{n}\right]\right\} & \left(\varepsilon \leq \varepsilon_{c}\right)\\ E\left(\varepsilon -\varepsilon_{c}\right)\left(1-D\right)+\sigma_{c} & \left(\varepsilon \geq \varepsilon_{c}\right) \end{array}\right.$$

where *b* and *n* are the scale parameter and shape parameter of the material in the void compression stage, *ε*_c_ is the strain corresponding to the end of the void compression stage, and *σ*_c_ is the stress corresponding to the end of the void compression stage.

Substituting Equation (6) into Equation (8) yields the constitutive equation of porous cement mortar:

(9) $$\sigma =\left\{\begin{array}{ll} E\varepsilon \left\{1-\exp\left[-{\left(\frac{\varepsilon }{b}\right)}^{n}\right]\right\} & \left(\varepsilon \leq \varepsilon_{c}\right)\\ E\left(\varepsilon -\varepsilon_{c}\right)\exp\left[-{\left(\frac{\varepsilon -\varepsilon_{c}}{a}\right)}^{m}\right]+\sigma_{c} & \left(\varepsilon \geq \varepsilon_{c}\right) \end{array}\right.$$

(10) $${\left.\frac{d\sigma }{d\varepsilon }\right|}_{\varepsilon =\varepsilon_{c}}=E$$

Meanwhile, the equation of the constitutive model meets the following conditions:

(11) $$\sigma \left|{}_{\varepsilon =\varepsilon_{m}}\right.=\sigma_{m}$$

(12) $${\left.\frac{d\sigma }{d\varepsilon }\right|}_{\varepsilon =\varepsilon_{m}}=0$$

(13) $$\sigma \left|{}_{\varepsilon =\varepsilon_{c}}\right.=\sigma_{c}$$

where *ε*_m_ is the peak strain, and *σ*_m_ is the peak stress of the material.

In this study, the initial slope of envelope curve *k* is introduced into Equation (9) to optimize the constitutive model, and the modified model can be expressed as

(14) $$\sigma =\left\{\begin{array}{ll} (E-k)\varepsilon \left\{1-\exp\left[-{\left(\frac{\varepsilon }{b}\right)}^{n}\right]\right\}+k\varepsilon & \left(\varepsilon \leq \varepsilon_{c}\right)\\ E\left(\varepsilon -\varepsilon_{c}\right)\left(1-D\right)+\sigma_{c} & \left(\varepsilon \geq \varepsilon_{c}\right) \end{array}\right.$$

where *k* is the initial slope of the envelope curve.

Combining Equations (9)–(13), *a* and *m* can be derived as follows:

(15) $$a=\left(\varepsilon_{m}-\varepsilon_{c}\right)m^{\frac{1}{m}}$$

(16) $$m={\left\{\ln\left[\frac{E\left(\varepsilon_{m}-\varepsilon_{c}\right)}{\sigma_{m}-\sigma_{c}}\right]\right\}}^{-1}$$

where *a* and *m* are the parameters of the Weibull distribution, so they are the same as the parameters in Equation (6). In Equation (6), the parameters are determined by the fitted curves obtained by several damaged variable points, while in Equations (15) and (16), *a* and *m* are determined by the boundary conditions of the envelope curves. Both approaches can determine the parameters and result in approximately equal parameters, so parameters *a* and *m* in the constitutive damage model in this paper are the average values obtained by these two approaches.

In the void compression stage, the constitutive model should be satisfied for Equation (10); thus, *b* and *n* can be derived as

(17) $$b=\varepsilon_{c}n^{\frac{1}{n}}$$

(18) $$n={\left\{-\ln\left[1-\frac{\sigma_{c}-k\varepsilon_{c}}{\varepsilon_{c}(E-k)}\right]\right\}}^{-1}$$

The parameters *b* and *n* of the constitutive damage model can be identified by substituting the data of cyclic uniaxial compression tests into Equations (17) and (18). As shown in Figure 10, the blue lines are the theoretical envelope curves of porous cement mortar, and the red lines are the experimental curves of the cyclic uniaxial compression tests. Table 4 lists the absolute errors between the envelope of the experimental stress–strain curve and the proposed theoretical model. In the pre-peak stages, the theoretical envelope curves obtained by the proposed model are almost the same as those of the experimental curves. Compared with the experiment results, the proposed theoretical model has an average absolute error of 0.54 MPa and a maximum absolute error of 0.99 MPa in the pre-peak stage. In the compaction stage, as the strain increases, the theoretical curves grow slowly from 0. After entering the elasticity stage, the slope of the theoretical curves dramatically increases to Young’s modulus and then gradually reduces. When the strain increases to the peak strain, the slope of the theoretical curves reduces to 0, and the theoretical curves reach the maximum value. In the post-peak stages, nonlinear strain softening occurs, and the experimental curves begin to decrease rapidly, resulting in the experimental curves lower than the theoretical curves. Compared with the experiment results, the proposed theoretical model has an average absolute error of 6.84 MPa and a maximum absolute error of 15.62 MPa in the post-peak stage. Thus, the proposed damage constitutive model can reflect the damaged mechanical properties of porous cement mortar in the compaction and pre-peak stages but cannot reflect the complex post-peak behaviors very well. The post-peak mechanical behavior of porous materials subjected to load is very complex, and most of the current mechanical models are difficult to accurately describe the post-peak stress–strain relationship of porous materials. The proposed damage constitutive model has a very large error after the peak because of the following reasons. First, the proposed model assumes that the damage evolution law follows the Weibull’s theory, which does not well reflect the post-peak damage evolution law of the porous mortar. Second, the mortar specimen is prone to fail suddenly after the peak, and the sudden failure could result in a rapid decline in the stress–strain curve. Such abrupt deformation behavior is difficult to be described by the constitutive damage model.

## 6. Conclusions

In this paper, based on the results of the cyclic uniaxial compression tests, experimental and theoretical analyses were conducted to study the compaction behavior and the mechanical damage evolution characteristics of porous cement mortar, and the following main conclusions were obtained:

For porous materials such as cement mortar, due to the larger aperture degree between particles, the mechanical properties are significantly different from other materials. We took the irreversible strain at the end of each loading step as a function of damage variable *D* for uniaxial tests.

Through this damage variable, the damage evolution model based on the Weibull distribution function is established, showing that the curves of the damage variable present an exponential trend and that most of the damage accumulates after the compaction stage.

The constitutive damage model of porous cement mortar is improved by considering both the damage evolution during the plastic deformation stage and the mechanical behavior in the compaction stage. The theoretical curves are in good agreement with the experimental envelope curve in the compaction and pre-peak stages.

The results of this study can provide a reference for the evaluation of the mechanical behavior of porous cement mortar and other similar materials under cyclic uniaxial compression conditions.

## Figures and Tables

**Table 1:** Basic physical parameters of the cement mortar specimens.

Name	Mass (g)	Density (g/cm^3^)	Longitudinal Wave Velocity (m/s)
cm-1	394.1	2.08	3594
cm-2	400.8	2.07	3508
cm-4	405.1	2.11	3485
cm-5	397.3	2.04	3540
cm-7	395.7	2.10	3526
cm-8	402.0	2.11	3578

**Table 2:** Peak strengths and Young’s modulus of different specimens.

Type of Tests	Name	Peak Stress (MPa)	Young’s Modulus (GPa)
Uniaxial compression tests	cm-1	36.13	10.23
	cm-2	35.86	10.50
	cm-4	37.08	9.89
	Mean value	36.35	10.21
Uniaxial cyclic loading and unloading tests	cm-5	35.52	9.89
	cm-7	35.31	8.64
	cm-8	36.61	7.80
	Mean value	35.81	8.78

**Table 3:** The accumulated irreversible strain during cyclic loading.

Cycle Number	Irreversible Strain (%)		
	cm-5	cm-7	cm-8
1	0.0478	0.0491	0.0529
2	0.0526	0.0604	0.0679
3	0.0616	0.0719	0.0797
4	0.0682	0.0836	0.0960
5	0.0779	0.1004	0.1135
6	0.0875	0.1161	0.1315
7	0.1003	0.1366	0.1532
8	0.1141	0.1610	0.1726
9	0.1395	0.1982	0.2032
10	0.1774	0.2711	0.2446
11	0.2618	0.3494	0.3210
12	-	-	0.4068

**Table 4:** The errors between the envelope of the experimental stress–strain curve and the introduced theoretical model.

Deformation Stage	Axial Strain (%)	Absolute Errors of Stress (MPa)				
		cm-5	cm-7	cm-8	Maximum Errors	Average Errors
Pre-peak	0.2	0.17	0.99	0.99	0.99	0.54
	0.4	0.81	0.77	0.02		
	0.6	0.01	0.17	0.95		
Post-peak	0.8	7.21	0.12	0.13	15.62	6.84
	1.0	12.21	1.74	3.36		
	1.2	10.26	15.62	10.95		

## Data Availability

The datasets used in the current study are available from the corresponding author upon reasonable request.
